# Association of clot ultrastructure with clot perviousness in stroke patients

**DOI:** 10.1038/s41598-023-41383-z

**Published:** 2023-09-04

**Authors:** Young Dae Kim, Il Kwon, Youngseon Park, Hyungwoo Lee, Il Hyung Lee, In Hwan Lim, Soon-Ho Hong, Hye Sun Lee, Hyo Suk Nam, Ji Hoe Heo

**Affiliations:** 1 Department of Neurology, Yonsei University College of Medicine, 50-1 Yonsei-ro, Seodaemoon-gu, Seoul, 03722 South Korea; 2 Integrative Research Institute for Cerebrovascular and Cardiovascular Diseases, Yonsei University College of Medicine, Seoul, South Korea; 3 Biostatistics Collaboration Unit, Department of Research Affairs, Yonsei University College of Medicine, Seoul, South Korea

**Keywords:** Neuroscience, Neurology

## Abstract

Clot perviousness on computerized tomography (CT) is predictive of response to reperfusion therapy. This study aimed to determine the association of clot perviousness with ultrastructural features of clot in stroke patients undergoing endovascular thrombectomy. We quantitatively analyzed the ultrastructural components identified using scanning electron microscopy. The clot components were determined in the inner portions of the clots. Clot perviousness was assessed as thrombus attenuation increase (TAI) using noncontrast CT and CT angiography. We compared the association between the identified ultrastructural components and clot perviousness. The proportion of pores consisted of 3.5% on scanning electron microscopy images. The proportion of porosity in the inner portion was 2.5%. Among the ultrastructural components, polyhedrocytes were most commonly observed. The mean TAI was 9.3 ± 10.0 (median 5.6, interquartile range 1.1–14.3) Hounsfield units. TAI correlated positively with inner porosity (r = 0.422, p = 0.020). Among the ultrastructural clot components, TAI was independently associated with polyhedrocytes (B = − 0.134, SE = 0.051, p = 0.008). Clot perviousness is associated with porosity and the proportion of polyhdrocytes of clots.

## Introduction

Thrombus is the end-product of pathological coagulation, which may produce vascular occlusion and clinical events^1^. After the implementation of endovascular treatment (EVT) for acute ischemic stroke, thrombi extracted from the intracranial artery can be obtained. Investigation of clots may provide opportunities to understand the pathophysiology and etiology of thrombosis and to gain insight into prognosis and treatment in stroke patients with EVT^2–4^.

Clot perviousness is quantified by the difference in the Hounsfield unit (HU) values between computed tomography (CT) angiography (CTA) and non-contrast CT (NCCT). Clot perviousness has been introduced as a potential imaging biomarker predicting recanalization outcomes after reperfusion therapy^5–7^. Previous studies have mainly focused on the association between clot perviousness and gross histologic composition of clots, and showed that clots with lower erythrocyte fractions are more permeable^6,8–11^. Clots have various ultrastructural features and the shape of erythrocytes is also very diverse on electron microscopy (EM)^2,12^. These ultrastructural features of clots including pores may be directly associated with clot perviousness^13,14^. However, little information is available on the relationship between ultrastructural features of clots and clot perviousness.

In this study, we investigated the ultrastructure of clots using scanning electron microscopy (SEM) and determined the association between quantitative morphological findings and clot perviousness on CT in stroke patients with EVT.

## Results

### Baseline characteristics

Thirty-nine consecutive patients had clots prepared for the SEM. Of these, 30 patients were included after excluding nine patients whose clots were not identified in the initial brain NCCT. The mean age was 68.4 ± 12.6 (median 70.5, interquartile range 56.8–79.3) years, and 56.7% patients were men (Table 1). The most common occlusion site was the middle cerebral artery (66.7%), followed by the distal internal carotid artery (26.7%) and basilar artery (6.7%). Stroke etiologies included cardioembolism (50%) and large artery atherosclerosis (50%). Intravenous thrombolysis was performed in 33.3% (n = 10) of the patients (Table 1).

**Table 1:** Baseline characteristics.

Variables	N (%) or mean ± SD
Age	68.4 ± 12.6
Sex, (male)	17 (56.7)
Hypertension	21 (70.0)
Diabetes	6 (20.0)
Dyslipidemia	8 (26.7)
Waist	84.0 (10.6)
Body mass index	23.1 (3.5)
Current smoking	8 (26.7)
Coronary artery occlusive diseases	2 (6.7)
Atrial fibrillation	14 (46.7)
Previous ischemic stroke	3 (10.0)
Active cancer	3 (10.0)
Previous antiplatelet	10 (33.3)
Previous oral anticoagulants	6 (20.0)
Previous statin	8 (26.7)
Non-cardioembolic stroke	15 (50.0)
Occlusion site	
Distal internal carotid artery	8 (26.7)
Middle cerebral artery	20 (66.7)
Basilar artery	2 (6.7)
Initial NIHSS	13.5 (8.0–19.0)
Intravenous t-PA	10 (33.3)
Laboratory variables	
White blood cell count, 10^9^/L	8056.3 ± 2533.5
Hemoglobin, g/L	13.5 ± 2.1
Platelet count, 10^3^/L	207.8 ± 66.5
Creatinine, µmol/L	76 ± 18.2
Glucose, mmol/L	7.6 ± 2
Treatment outcomes	
Duration of procedure, min	110.2 ± 191.1
TICI grade	
2b	8 (26.7)
3	22 (73.3)
Number of stent passage^a^	
1	12 (50.0)
2	7 (29.2)
≥ 3	5 (20.8)

Values are given as n (%), unless otherwise indicated.

SD, standard deviation; NIHSS, National Institutes of Health Stroke Scale; t-PA, tissue plasminogen activator; TICI, thrombolysis in cerebral infarction.

^a^Assessed in 24 patients treated with stentrievers.

### Ultrastructure of clots

The largest element of clots was polyhedrocytes (47.2%), followed by fibrin-platelet mixture (24.8%) and fibrin bundles (9.5%) (Supplementary Table 1). The proportion of pores was 2.5%. In comparison between the SEM and immunohistochemistry data for 23 patients with both data, the proportions of RBCs on immunohistochemistry were well correlated with those of polyhedrocyte (r = 0.455, p = 0.029) on SEM and that of fibrin on immunohistochemistry was correlated with fibrin bundle (r = 0.362, p = 0.090) (Table 2 and supplementary Fig. 1). However, the extent of platelets on SEM did not correlate with the proportion of platelets on immunohistochemistry (r = 0.048, p = 0.827).

**Table 2:** Correlation between findings of scanning electron microscopy and immunohistochemistry.

SEM	Immunohistochemistry						
	Platelet		Fibrinogen		RBC		
	r	p	r	p	r	p	
Fibrin fibers	0.430	0.041	0.117	0.594	− 0.327		0.127
Fibrin sponges	− 0.265	0.222	0.192	0.380	− 0.123		0.576
Fibrin bundles	0.263	0.226	0.362	0.090	− 0.196		0.370
Platelet	0.048	0.827	0.097	0.659	− 0.169		0.442
Fibrin-platelet mixture	− 0.002	0.993	− 0.360	0.091	− 0.061		0.781
Leukocyte	− 0.185	0.399	0.020	0.927	0.369		0.083
Concave RBC	0.204	0.351	0.278	0.199	− 0.304		0.158
Polyhedrocyte	− 0.158	0.471	− 0.048	0.826	0.455		0.029
Echinocyte	0.017	0.939	0.278	0.198	0.268		0.217
Balloon RBC	0.130	0.555	0.270	0.212	− 0.463		0.026
Intermediate RBC	0.310	0.149	0.331	0.123	− 0.160		0.467
Biofilm	0.303	0.159	0.364	0.087	− 0.292		0.176
Pore	0.327	0.128	0.281	0.194	− 0.409		0.053

SEM, scanning electron microscopy; RBC, red blood cell.

**Figure 1:**
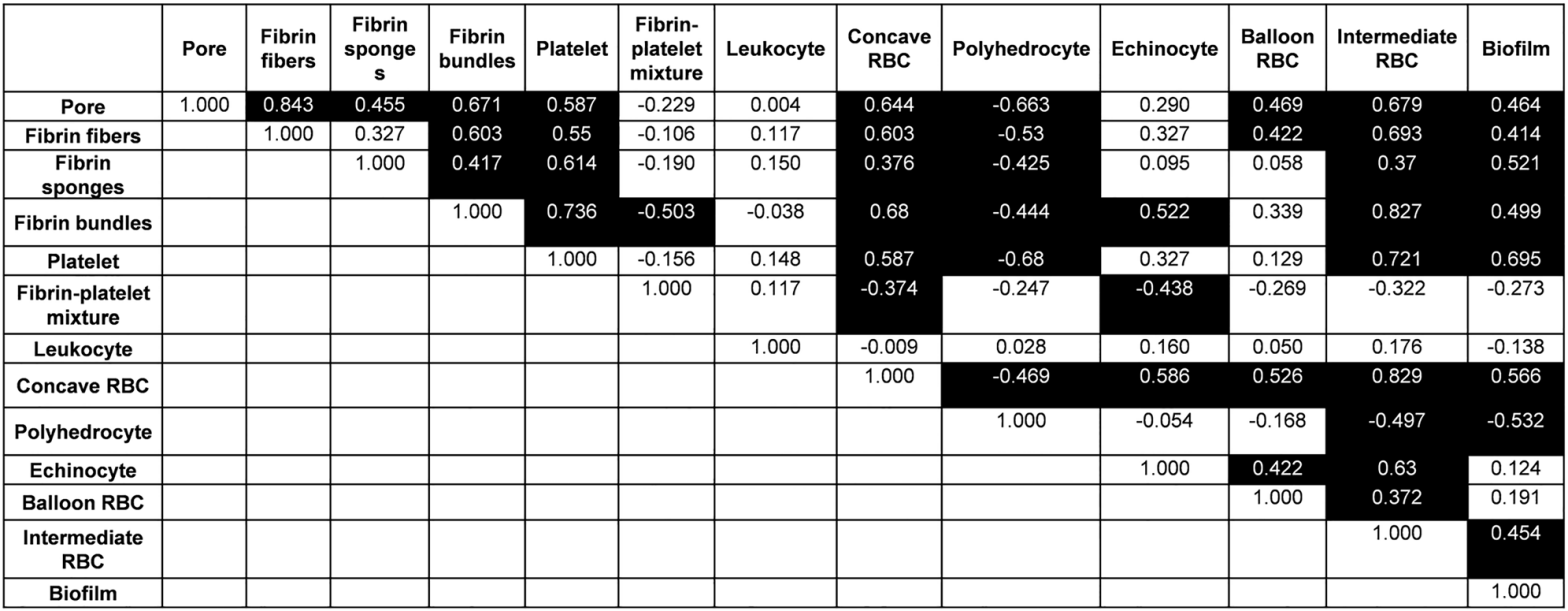
Spearman’s correlation between porosity and the ultrastructural clot components. Numbers are the correlation coefficients and black box indicates p < 0.05.

Although each clot element was intricately related to each other, porosity was associated with various clot elements. In particular, as the amount of fibrin increased or the amount of polyhedrocytes decreased, the porosity increased (Fig. 1). Among the clinical and laboratory variables, the hemoglobin level was also inversely related to porosity (r = − 0.379, p = 0.039).

### Relationship between clot perviousness and porosity

The mean thrombus attenuation increase (TAI) was 9.3 ± 10.0 (median 5.6, interquartile range 1.1–14.3) HU. TAI was positively correlated with porosity (r = 0.402, p = 0.028) (Table 3). In multivariable analysis adjusting the prior use of antiplatelet therapy, which was a significant factor for TAI in univariable analysis (p = 0.049) (Supplementary Table 2), TAI correlated positively with the amount of porosity (B = 1.777, SE = 0.434, p < 0.001).

**Table 3:** Spearman’s correlation between thrombus attenuation increase and ultrastructural clot components.

	r	p
Pore	0.402	0.028
Fibrin fibers	0.094	0.620
Fibrin sponges	0.291	0.118
Fibrin bundles	0.306	0.100
Platelet	0.234	0.213
Fibrin-platelet mixture	− 0.117	0.538
Leukocyte	0.277	0.138
Concave RBC	0.187	0.323
Polyhedrocyte	− 0.400	0.029
Echinocyte	− 0.038	0.844
Balloon RBC	0.247	0.189
Intermediate RBC	0.304	0.103
Biofilm	0.092	0.629

RBC, red blood cell.

### Relationship between clot perviousness and the cellular or non-cellular elements

Among the ultrastructural elements of clots, TAI increased as the amount of polyhedrocytes within clots decreased (r = − 0.400, p = 0.029) (Table 3). After adjustment of prior use of antiplatelet agents which was a significant factor for TAI in univariable analysis, TAI independently correlated with polyhedrocytes (B = − 0.134, SE = 0.051, p = 0.008). There was no association between recanalization outcomes and TAI or porosity (Supplementary Table 3).

## Discussion

This study demonstrated that clot perviousness is associated with the ultrastructure of clots. As the amount of inner porosity increased, the clot permeability increased. Among the ultrastructural elements of clots, polyhedrocytes were inversely related to clot porosity and were an independent determinant of clot perviousness.

The increased efficacy of reperfusion therapy may depend on the architecture of clots. Previously, clot perviousness has been proposed as a determinant for faster, successful recanalization and favorable functional outcome in reperfusion therapy^5–7,15^. The high permeability could be related to not only distal tissue oxygenation but also the more effective activation of endogenous plasminogen through the improved contact area, subsequently leading to the enhanced dissolution of fibrin filaments and clots^5–7,15,16^.

In this study, clot permeability was dependent on the clot ultrastructure, including porosity. In particular, the clot perviousness increased as the porosity increased. Large numbers of inner pores could result in wide and rapid penetration of the contrast agent within the clots. Further, porosity was positively associated with the amount of fibrin components (fibers, sponges, and fibrin bundles) and platelets in the inner portion of the clots. Previously, the histologic composition of clots, such as the amount of fibrin-platelets or RBCs, was suggested to be associated with clot perviousness^6,8–11^. Substantially, fibrin meshes possess some extent of empty space, which could allow easy penetration of contrast agents. The higher affinity of the fibrin proteins to iodine of contrast agents was speculated to be the possible reason for the association^17,18^. Our findings suggest that fibrin dominance of clots may indicate increased porosity within clots, and the increased porosity leads to increased permeability.

Hemoglobin level was also associated with the clot porosity. High hematocrit can elevate the blood viscosity and prothrombotic conditions. In addition, RBC can increase the platelet adhesion and aggregation, which involve the clot contraction, subsequently leading compacted RBC and impermeable clot. A previous study using human thrombus demonstrated that the clot with high RBC fraction was less responsive to intravenous thrombolysis^19^.

Moreover, among the ultrastructural components of clots, the number of polyhedrocytes was an independent predictor of clot perviousness. Polyhedrocytes are compressed erythrocytes resulting from the application of platelet-generated contractile or compressive force, and are a common form of RBCs in extracted clots^14,20^. Our results confirmed that polyhedrocytes were the most common form of RBCs within the clots (approximately 50%). It is biologically important that polyhedrocytes minimize the space between cells by more efficient packing. Packed polyhedrocytes help to create an impermeable seal at the site of vessel injury to prevent bleeding^21^. A previous report showed that clot perviousness negatively correlated with the percentage of RBCs in thrombi on hematoxylin and eosin stain^6^. In this study, the proportion of polyhedrocytes was correlated with the amount of RBCs on immunohistochemistry. Our findings suggest that the association between RBCs in clots and clot perviousness is mainly due to the presence and amount of polyhedrocytes.

This study has several limitations. The thrombi used were extracted from patients with successful recanalization. Additionally, although assessments were extensive in many randomly selected areas of thrombi, thrombus samples for SEM might not have been representative of whole clots. In fact, although the proportion of some components on SEM were not correlated with those in immunohistochemistry. Finally, the sample size was small; therefore, we were unable to evaluate the clinical and laboratory factors that were associated with different ultrastructural characteristics among patients.

In conclusion, clot perviousness was associated with porosity or the number of polyhdrocytes.

## Methods

### Study population

This retrospective study used registry data and thrombi obtained from patients who had undergone EVT with large intracranial cerebral artery occlusion between September 2017 and May 2020 at the Severance Stroke Center. Clinical data were derived from the prospective Specialized Multi-center Attributed Registry of Stroke-Endovascular or Thrombolytic Therapy (SMART-EST) registry (Clinicaltrials.gov NCT04066556). The SMART-EST is a prospective multicenter registry for consecutive patients with acute ischemic stroke who have received the standard reperfusion therapy since July 2019 in 22 hospitals in South Korea. The retrieved thrombi were primarily used for immunohistochemistry. This study included patients with available thrombi for EM and thin-section CT and CTA of the brain for the evaluation of clot perviousness. This study was approved by the Institutional Review Board of Yonsei University College of Medicine (No. 4-2022-1268). Written informed consent was obtained from patients or the next of kin. The present study was performed in accordance with the relevant guidelines and regulations.

Reperfusion therapy was performed using protocols based on the guidelines. We collected demographic, medical or laboratory, and imaging data. Detailed information is provided in the Supplementary Methods.

### Imaging and clot perviousness assessment

Patients underwent NCCT and CTA at baseline using a multidetector-row CT scanner (Light Speed Plus; GE Medical Systems, Piscataway, NJ, USA; Sensation 16, Siemens Medical Systems, Brilliance 64, Philips Medical Systems). Baseline NCCT included both a 5-mm and either a 1.25-mm or 1-mm thin-section slice according to a standardized protocol (Supplementary Methods).

Clot perviousness was assessed by contrast agent uptake in CTA images, measured as the increase in Hounsfield units (HUs) between the NCCT and CTA scans. Two raters (YDK and SHH) who were blinded to all clinical data measured the HU. The measurement of clot perviousness was based on previous reports^9^. Briefly, we measured the HU by manually placing spherical regions of interest (ROIs) with a 1-mm radius within the clot on both NCCT and CTA images. CTA images were read side-by-side within the picture archiving and communication system to ensure accurate placement of ROIs within the clot location on the NCCT images. Up to five ROIs were placed over each clot, whereas two ROIs were used for smaller thrombi. If multiple ROIs were obtained, the mean value of measured HUs was used. Thereafter, the TAI was calculated, which was defined at the increase in the mean attenuation of the thrombus on CTA compared with NCCT.

### High-resolution SEM of clots

While minimizing mechanical perturbation, the retrieved thrombi were rinsed with saline and fixed for 24 h in Karnovsky's fixative (2% glutaraldehyde, 2% Paraformaldehyde in 0.1 M phosphate buffer, pH 7.4). Following fixation, the samples were cut open longitudinally so that the interior parts, edges, and surfaces of the clots could be viewed. The fixed cerebral thrombi were washed in 0.1 M phosphate buffer (pH 7.4), dehydrated in ascending concentrations of ethanol (50–100 v/v%), dried using a critical point dryer (Leica EM CPD300, Leica Microsystems, Wetzla, Germany), and coated with platinum by ion sputtering (Leica EM ACE600, Leica Microsystems). High-resolution micrographs were obtained from randomly selected 10 areas between the edge and center of the clots from the interior parts of the thrombi to eliminate selection bias and characterize their overall composition (Supplementary Fig. 2). Images were obtained using a field SEM (Merlin; Zeiss, Oberkochen, Germany).

### Quantification of clot composition using SEM

**Figure 2:**
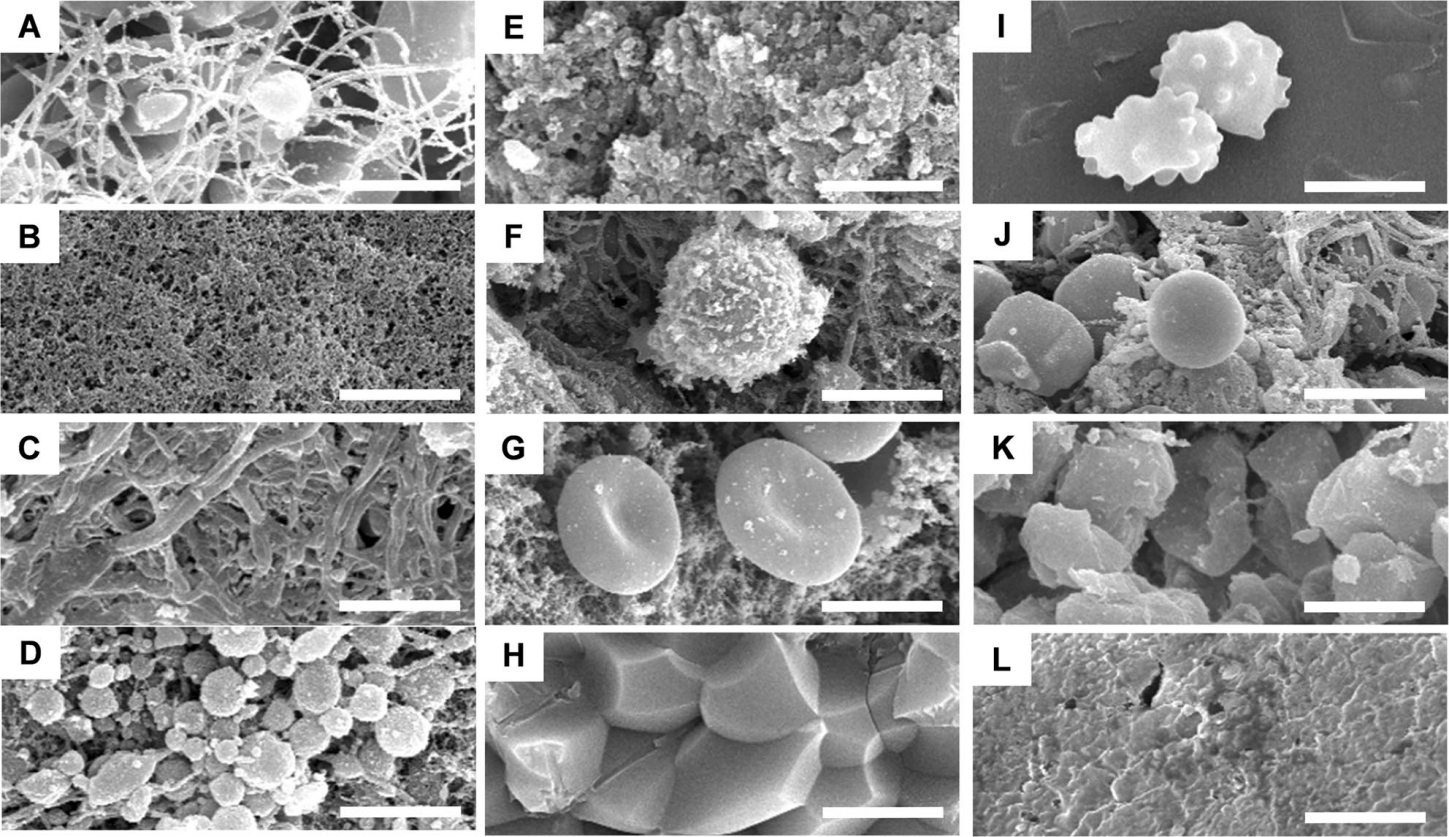
Components of clots detected in scanning electron micrographs. (**A**) Fibrin fiber; (**B**) fibrin sponge; (**C**) fibrin bundle; (**D**) platelet; (**E**) fibrin/platelet mixture; (**F**) leukocyte; (**G**) concave red blood cells (RBCs); (**H**) polyhedrocytes; (**I**) echinocytes; (**J**) balloon RBCs; (**K**) intermediate RBCs; (**L**) biofilm. Magnification bar = 5 μm.

Thrombi were analyzed quantitatively for their elements using SEM, which was based on a previous study^12^. First, various ultrastructural elements were identified by SEM (Fig. 2). Red blood cells (RBCs) were segregated into five morphological groups: concave RBC, polyhedrocytes, echinocytes, balloon RBC, and intermediate RBC. Fibrin is categorized into three morphological types: fibrin fibers, fibrin sponges, and fibrin bundles. Platelet and leukocyte were also determined. When fibrin and platelet aggregates were mixed and resembled a coarse mud wall, we defined it as a fibrin-platelet mixture. Additionally, we assessed the amount of biofilm that looked like a membrane. Empty spaces between the above-mentioned structures were defined as pores. Detailed descriptions of each cellular element are presented in Fig. 2 and Supplementary Table 4.

The element of the cerebral thrombi was quantified manually using PhotoQuad software^22^. The images were transposed onto a computer screen and a fine grid (grid squares 1 × 1 μm). The images were overlaid onto the scanning electron micrographs and each square of the grid was marked for the element(s) that it contained (Supplementary Fig. 3). A total of 600 images of 30 patients (20 images per patient) were obtained, and 2352 cells (56 × 42 cells) per image were overlaid. While counting the cells in a clot, we also counted the number of each cell type. Two observers (YDK and IK) counted each clot element. In summary, total image areas of approximately 740,880 μm^2^ from thrombi were quantified.

### Immunohistochemistry

The other fragments of retrieved thrombi were immediately fixed in 4% paraformaldehyde, embedded in paraffin blocks, and stored until use. The blocks were sectioned into 3-μm slices, deparaffinized, and rehydrated. The sections for CD42b were subjected to heat-induced epitope retrieval using the IHC-Tek epitope retrieval solution and steamer (IHC World, Inc., Woodstock, MD, USA). The primary antibodies used were anti-glycophorin A (ab129024, 1:400; Abcam, Cambridge, UK) for RBC, anti-CD42b (ab134087, 1:100; Abcam) for platelets, and anti-fibrinogen (ab34269, 1:200; Abcam) for fibrin/fibrinogen. The primary antibodies were incubated overnight at 4 °C, and goat anti-rabbit IgG antibody (BA-1000, Vector Laboratories, Burlingame, CA, USA) was used as a secondary antibody for all primary antibodies. Peroxidase signal was developed using a 3,3′-diaminobenzidine solution (D5637; Sigma-Aldrich, St. Louis, MO, USA).

Images of stained thrombi were scanned using a digital scanner (Aperio AT2; Leica Biosystems, Wetzlar, Germany). The scanned images were analyzed using Automated Region-of-interest based Image Analysis for automated composition analysis^23^. The auto threshold of pixel density was used. The fractions of glycophorin A-, CD42b-, and fibrin-positive areas were calculated as the percentage pixel density of the total thrombus area. The proportions of RBC, fibrin/fibrinogen, and platelet on immunohistochemistry stain was compared with the proportions of each component observed in SEM.

### Statistical analyses

Statistical analyses were performed using the Windows IBM SPSS software package (version 21.0, Chicago, IL, USA) or the R software package version 3.0.1 (http://www.R-project.org). Continuous variables were compared using Mann–Whitney tests, Kruskal–Wallis tests, or the paired Wilcoxon signed-rank test, while categorical variables were compared using chi-square tests or Fisher’s exact tests, as appropriate. The correlation between each clot component was investigated using Spearman’s correlation analysis. The relationship between the change in HU of clots according to clot characteristics was investigated using Tobit regression analysis to compute the odds ratios of covariates. Statistical significance was set at p < 0.05.

### Supplementary Information

Supplementary Information.

## Data Availability

The datasets generated and analysed during the current study are available from the corresponding author on reasonable request.

## References

[1] Kappelhof M (2021). Endovascular treatment effect diminishes with increasing thrombus perviousness: Pooled data from 7 trials on acute ischemic stroke. Stroke.

[2] Chernysh IN (2020). The distinctive structure and composition of arterial and venous thrombi and pulmonary emboli. Sci. Rep..

[3] Laridan E (2017). Neutrophil extracellular traps in ischemic stroke thrombi. Ann. Neurol..

[4] Heo JH (2020). Pathophysiologic and therapeutic perspectives based on thrombus histology in stroke. J. Stroke.

[5] Santos EM (2016). Thrombus permeability is associated with improved functional outcome and recanalization in patients with ischemic stroke. Stroke.

[6] Patel TR (2021). Increased perviousness on CT for acute ischemic stroke is associated with fibrin/platelet-rich clots. AJNR Am. J. Neuroradiol..

[7] Diamond SL, Anand S (1993). Inner clot diffusion and permeation during fibrinolysis. Biophys. J..

[8] Berndt M (2018). Thrombus permeability in admission computed tomographic imaging indicates stroke pathogenesis based on thrombus histology. Stroke.

[9] Benson JC (2020). Clot permeability and histopathology: Is a clot's perviousness on CT imaging correlated with its histologic composition?. J. Neurointerv. Surg..

[10] Borggrefe J (2018). Differentiation of clot composition using conventional and dual-energy computed tomography. Clin. Neuroradiol..

[11] Ye G (2021). Histological composition behind CT-based thrombus density and perviousness in acute ischemic stroke. Clin. Neurol. Neurosurg..

[12] Khismatullin RR (2020). Quantitative morphology of cerebral thrombi related to intravital contraction and clinical features of ischemic stroke. Stroke.

[13] Cines DB (2014). Clot contraction: Compression of erythrocytes into tightly packed polyhedra and redistribution of platelets and fibrin. Blood.

[14] Tutwiler V (2018). Shape changes of erythrocytes during blood clot contraction and the structure of polyhedrocytes. Sci. Rep..

[15] Santos EM (2016). Permeable thrombi are associated with higher intravenous recombinant tissue-type plasminogen activator treatment success in patients with acute ischemic stroke. Stroke.

[16] Ahn SH (2015). Occult anterograde flow is an under-recognized but crucial predictor of early recanalization with intravenous tissue-type plasminogen activator. Stroke.

[17] McDonald MM (2014). Iodinated contrast does not alter clotting dynamics in acute ischemic stroke as measured by thromboelastography. Stroke.

[18] Hertig G (2017). Iodixanol as a contrast agent in a fibrin hydrogel for endodontic applications. Front. Physiol..

[19] Choi MH (2018). Erythrocyte fraction within retrieved thrombi contributes to thrombolytic response in acute ischemic stroke. Stroke.

[20] Tutwiler V (2017). Contraction of blood clots is impaired in acute ischemic stroke. Arterioscler. Thromb. Vasc. Biol..

[21] Johnson S (2020). Mechanical behavior of in vitro blood clots and the implications for acute ischemic stroke treatment. J. Neurointerv. Surg..

[22] Trygonis V, Sini M (2012). photoQuad: A dedicated seabed image processing software, and a comparative error analysis of four photoquadrat methods. J. Exp. Mar. Biol. Ecol..

[23] Heo J (2022). Automated composition analysis of thrombus from endovascular treatment in acute ischemic stroke using computer vision. J. Stroke.

