# Supplementary material for: Association of clot ultrastructure with clot perviousness in stroke patients
Source: Sci Rep. 2023 Sep 4;13:14568. doi: 10.1038/s41598-023-41383-z (PMC10477321; doi:10.1038/s41598-023-41383-z)
Supplement: Supplementary file 1 — Supplementary Information. [file 41598_2023_41383_MOESM1_ESM.pdf]

Supplementary Materials

Association of clot ultrastructure with clot perviousness in stroke patients

Young Dae Kim, MD, PhD, IL Kwon, PhD, et al

List of items

Supplementary methods
Supplementary Figure 1. A representative case showing images of scanning electron microscopy and immunohistochemistry
Supplementary Figure 2. Ten randomly selected areas in inner portion of a thrombus
Supplementary Figure 3. Quantitative analysis overlaying the fine grid
Supplementary Table 1. Proportions of each component of clots
Supplementary Table 2. Univariable analysis of thrombus attenuation increase
Supplementary Table 3. Univariable analysis of porosity
Supplementary Table 4. Ultrastructural thrombus components collected in this study

Supplementary methods

Reperfusion therapy protocols

Endovascular therapy (EVT) was administered to patients who had large vessel occlusion up to 24 h after stroke onset. If patients arrived within 4.5 hours after stroke onset, he/she was treated with IV t-PA (Actylase; Boehringer-Ingelheim, Ingelheim, Germany) at a standard dose of 0.9 mg/kg (10% as bolus and then 90% as infusion for 60 min). EVT is performed primarily using mechanical devices rather than chemical agents. Among the mechanical devices, the Solitaire stent retriever, Trevo retriever, and Penumbra reperfusion catheter are available in Korea and were used based on the target vessel site, tortuosity, or neurointerventionalist preference. Intra-arterial thrombolysis with urokinase (Green Cross, Seoul, Korea) or glycoprotein IIb/IIIa antagonists was used as adjuvant therapy in certain cases, including those with re-occlusion or distal embolization. If the symptom onset was unclear, EVT was performed based on the imaging findings and the physician's discretion. Brain magnetic resonance imaging (MRI) and magnetic resonance angiography were performed 24 h after reperfusion therapy. When brain MRI could not be performed, brain computed tomography (CT) and/or CT angiography (CTA) were performed.

Clinical variables

We collected the patients' data in the following categories: vascular risk factors, including hypertension, diabetes mellitus, hyperlipidemia, and current smoking (any cigarette smoking within 1 year prior to admission); comorbidities, such as atrial fibrillation, previous coronary artery occlusive disease, history of prior medication, occlusion site of large vessel at index stroke, and the use of intravenous tissue plasminogen activator. Laboratory findings, including hemoglobin levels, white blood cell counts, platelet counts, and serum creatinine and glucose levels, were also recorded. Stroke severity was assessed using the National Institutes of Health Stroke Scale.

We also determined the duration of the procedure (intervals from femoral puncture to the end of EVT) and the number of stent passages if a stentriever was used. Successful recanalization, defined as thrombolysis in cerebral infarction grade 2b or 3 on the final digital subtraction angiography, was also collected.

Imaging protocols

Conventional axial 5-mm non-contrast CT (NCCT) was performed first, with the following parameters:120 kVp; 250 mA; rotation time, 0.8 s; and FOV, 25 cm. Overall, 28–30 images were obtained per examination, according to the size of the patient's head. Helical 1.25-mm or 1-mm NCCT was also obtained after 5-mm CT, with the following parameters:120 kVp; 250 mA; rotation time, 0.8 s; pitch, 0.75; FOV, 25 cm; matrix, 512 × 512; and pixel size, 0.49 × 0.49 mm. Scans were obtained parallel to the plane of the inferior orbital rim to the basin and ended 7.5 cm above it to reduce the radiation dose. All images obtained via 1.25-mm or 1-mm NCCT were reconstructed at a 0.6-mm thickness. CTA was performed with the same parameters as those used for helical 1.25-mm or 1-mm NCCT.

Supplementary Figure 1. A representative case showing images of scanning electron microscopy and immunohistochemistry. A clot was mainly consisted of the polyhedrocyte (91.8%), and the fibrin bundle (arrowheads) or fibrin-platelet mixture (arrows) was also found on scanning electron microscopy (A). Red blood cells (RBCs) were also the major component of clot on immunohistochemistry (B).

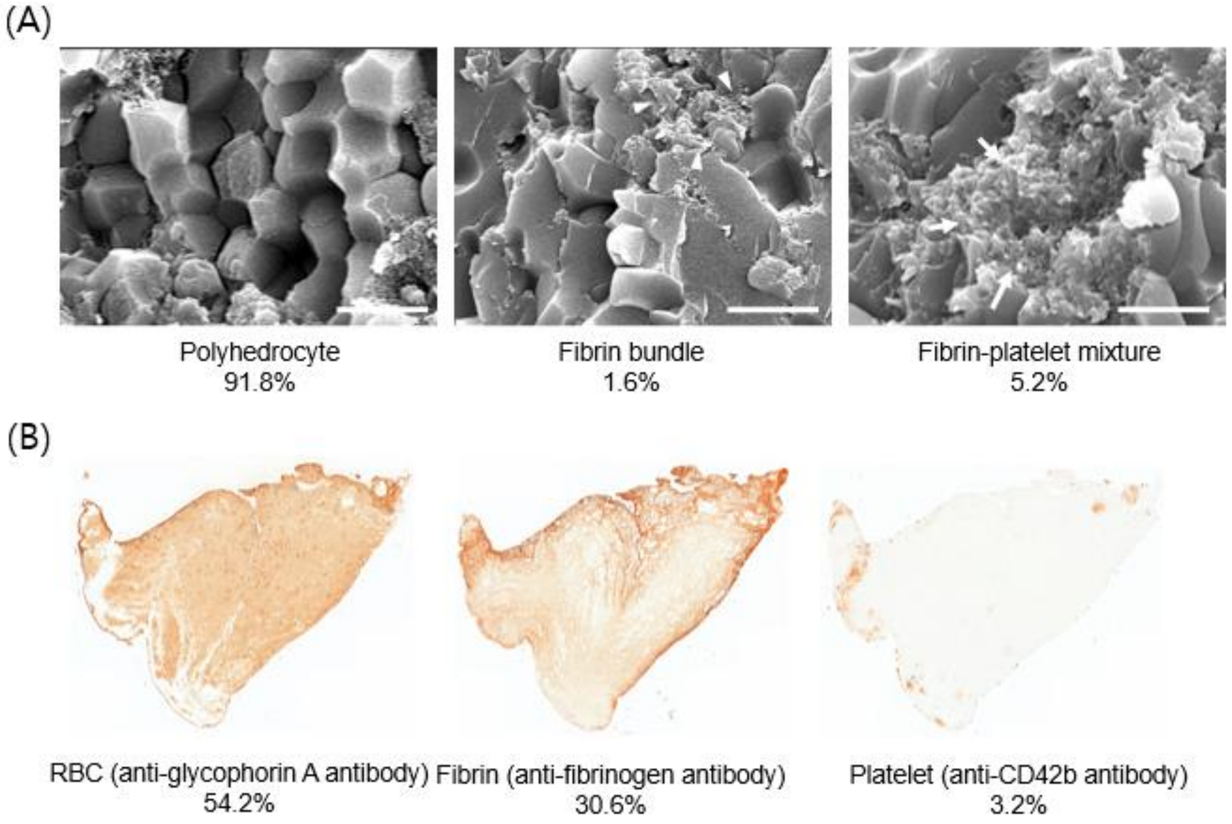

Supplementary Figure 2. Ten randomly selected areas in inner portion of a thrombus The blue circles indicate the selective area for collecting data on the components of the thrombus. Magnification bar = 500 μm

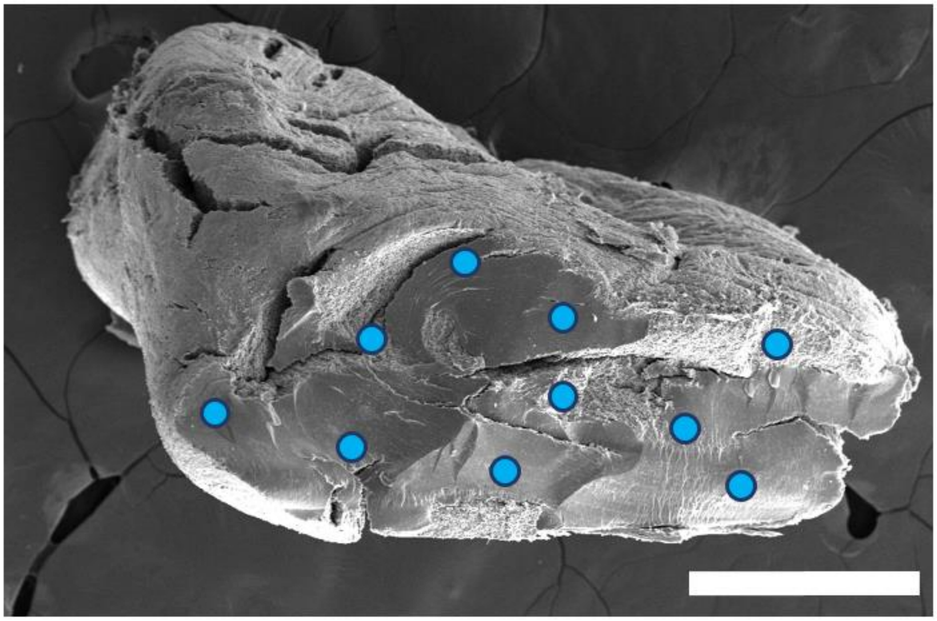

Supplementary Figure 3. Quantitative analysis overlaying the fine grid (grid squares 1 × 1 µm) Magnification bar = 5 µm

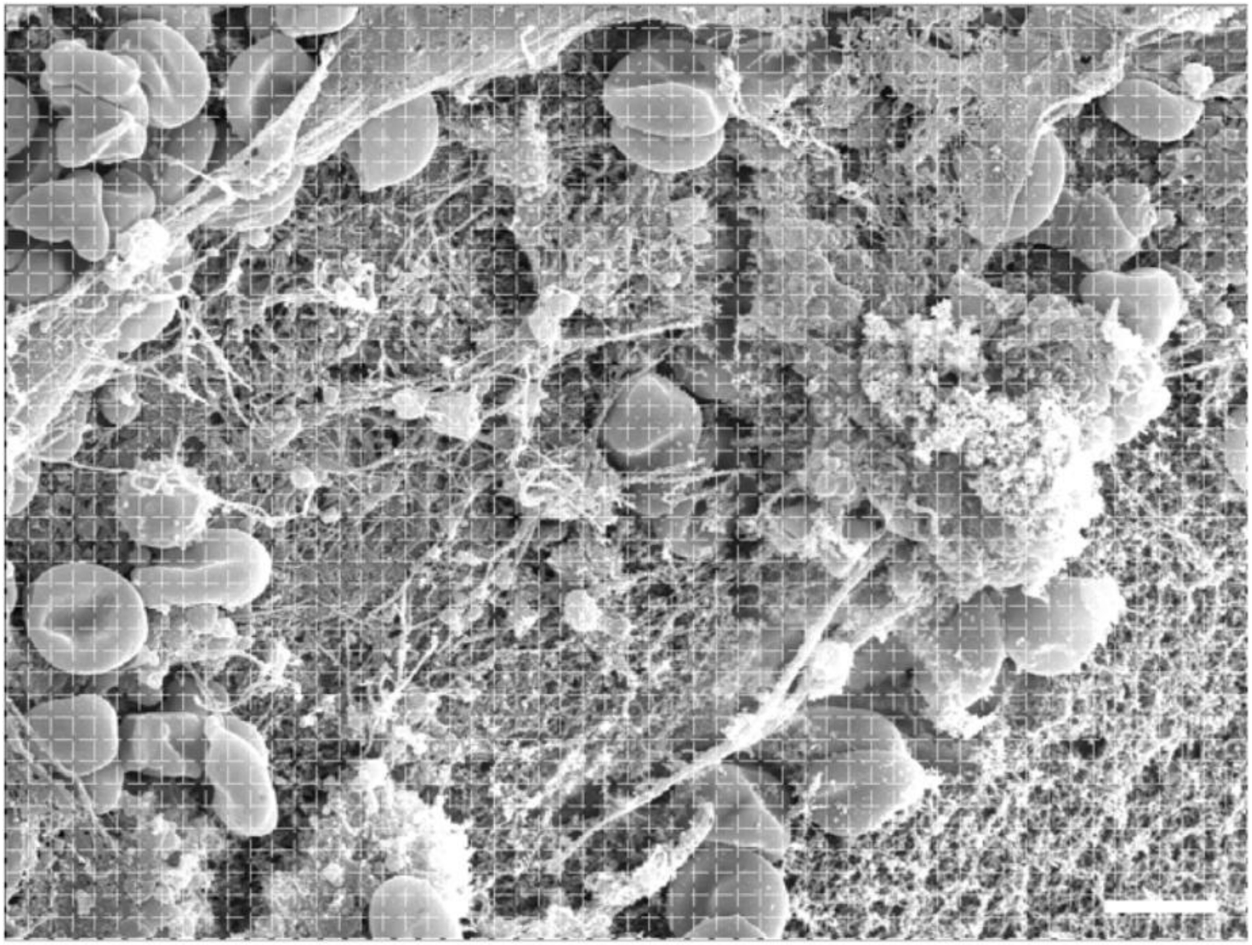

Supplementary Table 1. Proportions of each component of clots

	Mean ± SD	Median (IQR)
Fibrin fibers	5.3 ± 6.1	2.6 (1.0 - 9.2)
Fibrin sponges	1.0 ± 2.3	0.0 (0.0 - 1)
Fibrin bundles	9.5 ± 10.8	4.5 (2.1 - 14.4)
Platelet	3.0 ± 5.9	0.8 (0.1 - 2.2)
Fibrin-platelet mixture	24.6 ± 22.6	23.5 (4.7 - 37.8)
Leukocyte	0.3 ± 0.4	0.2 (0.0 - 0.4)
Concave RBC	0.5 ± 1.1	0.1 (0.0 - 0.3)
Polyhedrocyte	47.2 ± 30.4	52.4 (18.5 - 74.6)
Echinocyte	0.3 ± 0.6	0.0 (0.0 - 0.2)
Balloon RBC	0.1 ± 0.2	0.0 (0.0 - 0.2)
Intermediate RBC	1.8 ± 3.1	0.6 (0.0 - 1.7)
Biofilm	3.9 ± 8.7	0.0 (0.0 - 2.9)
Pore	2.5 ± 3.2	1.5 (0.6 - 3.4)

Supplementary Table 2. Univariable analysis of thrombus attenuation increase

Variables	B	SE	p
Age	0.254	0.154	0.1
Sex, (Male)	-0.451	4.007	0.91
Hypertension	6.222	4.154	0.134
Diabetes	4.569	4.839	0.345
Dyslipidemia	6.44	4.278	0.132
Waist	-0.046	0.207	0.825
Body mass index	0.178	0.574	0.756
Current smoking	-0.329	4.439	0.941
Coronary artery occlusive diseases	-10.93	8.556	0.201
Atrial fibrillation	6.165	3.829	0.107
Previous ischemic stroke	0.607	6.525	0.926
Active cancer	5.276	6.456	0.414
Prior use of antiplatelet	7.664	3.909	0.049
Prior use of oral anticoagulants	0.811	4.903	0.869
Prior use of statin	1.322	4.44	0.766
Non-cardioembolic stroke	-7.225	3.769	0.055
Occlusion site			
distal internal carotid artery	-5.962	4.343	0.17
middle cerebral artery	4.668	4.096	0.252
basilar artery	1.89	7.838	0.809
Initial NIHSS	-0.38	0.342	0.267
Intravenous t-PA	4.806	4.082	0.239
Laboratory variables			
White blood cell count, 10^9/L	-0.001	0.001	0.463
Hemoglobin, g/L	-0.688	0.943	0.466
Platelet count, 10^3/L	-0.015	0.03	0.627
Creatinine, µmol/L	0.137	0.11	0.213
Glucose, mmol/L	0.977	1.009	0.333
Treatment outcomes			
Duration of procedure, min	-0.008	0.01	0.45
TICI grade of 3 vs. 2b	4.211	8.821	0.633
Number of stent passage (n=24) of $\geq$2 vs. 1	3.871	4.514	0.391

NIHSS, National Institutes of Health Stroke Scale; t-PA, tissue plasminogen activator; TICI, thrombolysis in cerebral infarction.

Supplementary Table 3. Univariable analysis of porosity

	B (SE)	P
Duration of procedure, min	-0.001 (0.003)	0.657
TICI grade of 3 vs. 2b	0.917 (1.453)	0.528
Number of stent passage of ≥2 (vs. 1)*	0.917 (1.453)	0.528

TICI, thrombolysis in cerebral infarction.

* Assessed in 24 patients treated with stentrievers.

Supplementary Table 4. Ultrastructural thrombus components collected in this study

Components	Characteristics
**Red blood cell (RBC)**	
Concave RBC	both sides of the surface curve inward forming a dimple with size of 4- to 5-umcells
Polyhedrocyte	Polyhedral cells with surfaces comprising intersecting polygonal sides
Balloon RBC	Convex-shaped RBCs 5 μm in diameter with a smooth round surface without protrusions, indentations, or dimples
Echinocytes	RBC with multiple, small, evenly spaced thorny projections
Intermediate RBC	Various form of RBC not like concave RBC, polyhedrocyte, balloon RBC, or echinocyte
**Fibrin**	
Fibrin fibers	Thin fibrillar structures single or usually arranged into a network, The size of fibrin is 20 μm to 400 μm (0.02 μm to 0.4 μm)
Fibrin sponge	Amorphous structure or fibrin network composed of very fine fibers, often with bound platelets and microvesicles
Fibrin bundles	Thick fibrillar structures made up of several laterally aggregated fibers
**Platelet**	A various form of platelets were included; from round shape (<2–3 μm) to balloon platelet (multiple wrinkles and lines on the surface and no bumps with size of 4–7 μm) or platelet aggregates (Clusters of deformed irregular-shaped platelets with outgrowths and multiple filopodia)
**Fibrin/platelet mixture**	Mixture composing of collection of granular structure, irregular surface and tough texture
**Leukocytes**	Spherical or irregular-shaped cells with membrane folding and a rough surface with multiple short bumps (size of 5–12 μm)
**Biofilm**	Dense and smooth membrane or layer covering the structure
**Pore**	Empty spaces between the above mentioned structures
